# [2+2+2] Cycloaddition Reactions of Macrocyclic Systems Catalyzed by Transition Metals. A Review

**DOI:** 10.3390/molecules15129230

**Published:** 2010-12-15

**Authors:** Anna Pla-Quintana, Anna Roglans

**Affiliations:** Departament de Química, Universitat de Girona, E-17071-Girona, Spain

**Keywords:** azamacrocycles, alkynes, alkenes, [2+2+2] cycloadditions, transition metals

## Abstract

Polyalkyne and enediyne azamacrocycles are prepared from arenesulfonamides and various alkyne and alkene derivatives either under basic or neutral conditions. The new family of macrocyclic substrates is tested in the [2+2+2] cycloaddition reaction. Several catalysts are used for the cycloisomerization reaction, and their enantioinduction is evaluated as appropriate. The effect of the structural features of the macrocycles, namely the ring size, substituents in precise positions and the number and type of unsaturations, on the [2+2+2] cycloaddition reaction has also been studied.

## 1. Introduction

**Scheme 1:**
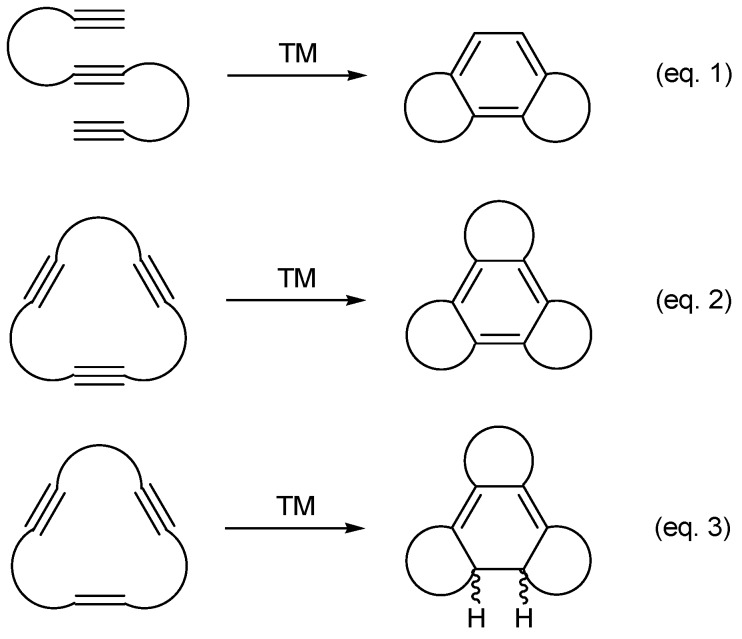
General representation of intramolecular [2+2+2] cycloadditions of triynes and enediynes.

A highly reliable and atom-economical method for the synthesis of polysubstituted benzene derivatives is the transition-metal-catalyzed [2+2+2] cycloaddition of three alkynes [1,2,3,4,5,6,7,8,9,10,11,12,13]. Completely intramolecular reactions are particularly interesting as they provide complex polycyclic systems in a single synthetic operation (Equation 1, Scheme 1) [14,15,16,17,18,19,20,21,22,23,24,25,26]. Furthermore, if the three alkynes form part of a closed system, *i.e.* a macrocycle, fused tetracycles may easily be obtained (Equation 2, Scheme 1). The [2+2+2] cycloaddition is not a reaction that is exclusive for triple bonds, as it can also take place between two acetylenes and one alkene giving rise to cyclohexadienes. Given this, the same concept could be applied for the completely intramolecular [2+2+2] cycloaddition of enediynes to afford fused polysubstituted cyclohexadienes (Equation 3, Scheme 1) [27,28,29,30,31,32,33].

**Scheme 2:**
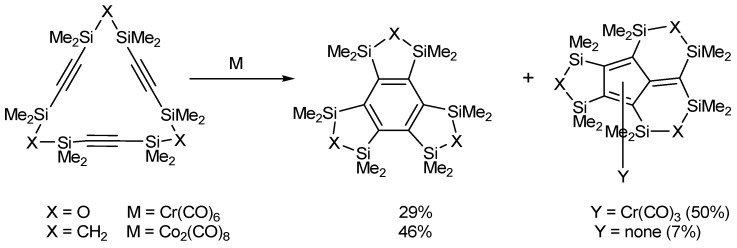
Intramolecular cyclization of macrocyclic polyacetylenes tethered by disiloxane and disilmethylene bridges.

Despite this strategy being synthetically attractive, hardly any cyclotrimerization reactions of macrocyclic systems containing triple bonds have been reported. Vollhardt, in an article published in 1976 [34], prepared 1,5,9-cyclododecatriyne, which proved to be inert in the presence of light, high pressure and temperature, acidic conditions, and the CpCo(CO)_2 _catalyst. The cycloisomerization of cyclotriynes into tricyclic benzene derivatives has only been reported in two silicon-tethered macrocycles by Sakurai *et al.* in low yields and in the presence of other π electron systems [35,36,37] (Scheme 2).

In recent years our group has been interested in the synthesis and applications of polyalkyne and enediyne azamacrocyclic systems [38,39,40,41,42,43,44]. Early on we observed that the [2+2+2] cycloisomerization of the closed derivatives could easily result in highly functionalized tetracyclic fused structures in one-pot and atom-economical process. In addition, since our macrocyclic systems contain nitrogen atoms in the tethers between unsaturations, their [2+2+2] cycloaddition reaction opens the door to the construction of polycyclic azaheterocycles.

In this review we present our work in this field, starting with highly efficient synthetic strategies towards several polyunsaturated azamacrocycles and then look at their cycloaddition reactions catalyzed by transition metals to afford fused polycyclic structures.

## 2. Synthesis of Polyunsaturated Azamacrocycles

### 2.1. Preparation of tri-, tetra-, and pentaacetylenic azamacrocycles

**Figure 1:**
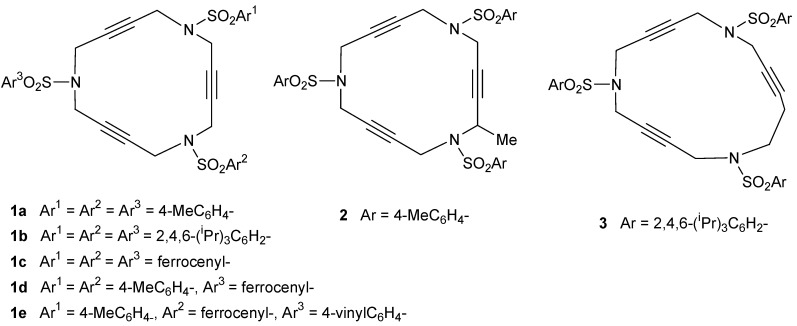
Triacetylenic azamacrocycles.

In a previous project in which we participated, synthetic routes towards 15-membered triazatrienic macrocycles were optimized [45,46]. This was our source of inspiration towards the synthesis of the array of triacetylenic macrocycles that have been prepared for testing in the [2+2+2] cycloaddition reaction, shown in Figure 1.

Different 15-membered azamacrocycles of type **1** were prepared by varying the nature of the arylsulfonamide to confer, enhance or modulate certain properties of the macrocycles. As an example, 4-methylphenyl aryl groups in the sulfonamide moiety give crystallinity to the macrocycles permitting X-ray diffraction analysis. Ferrocenyl groups confer an orange colour to the compounds facilitating their purification by column chromatography. The three isopropyl chains in the 2,4,6-triisopropylsulfonyl moiety improve solubility in standard organic solvents.

**Scheme 3:**
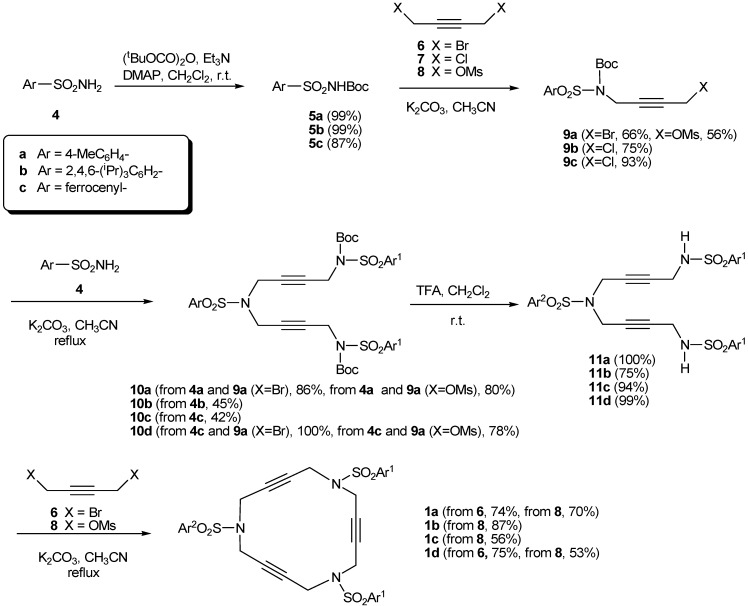
Synthesis of macrocycles **1a**, **1b**, **1c** and **1d**.

The synthesis of these 15-membered macrocycles is quite straightforward using sulfonamide alkylation reactions combined with N-*tert*-butyloxycarbonyl (Boc) protection/deprotection to direct the reaction to the desired product (control of mono- and dialkylation). The starting materials are commercially available arylsulfonamides and various 1,4-dihalo-2-butynes or derivatives. The synthetic routes varied depending on whether the arylsulfonamides contained in the azamacrocycle were the same or different. For macrocycles **1a-d**, which contain three identical arylsulfonamides (**1a** [38], **1b** [39], and **1c** [39]) or two identical and one different arylsulfonamide (**1d** [38]), the synthetic pathways are represented in Scheme 3. The synthesis started with arylsulfonamides **4a-c** which were protected by Boc to give the corresponding derivatives **5a-c**. Boc-protected sulfonamides **5a-c **were treated either with dibromide **6**, dichloride **7** or dimesylate **8** (4 equiv.) to afford derivatives **9a-c**.

The choice between **6**, **7** or **8 **depends on several factors. Firstly, on their availability: whereas the chloro derivative **7** is commercially available, bromo derivative **6 **and dimesylate **8** have to be specially prepared. Secondly, the yield of the reaction varies depending on the leaving group: as expected, best results are invariably obtained with bromo derivative **6** rather than the chloro derivative. The dimesylate **8** is quite effective, but thick salts are sometimes formed requiring mechanical stirring of the reaction mixtures. Finally, the powerful irritating properties of dibromo derivative **6** can cause a skin allergy and so extreme precaution has to be taken in the manipulation of both the product itself and associated waste products.

Once the Boc-protected arylsulfonamide was monoalkylated, the synthetic route continued with condensation of **9** with arylsulfonamides **4** to lead to **10**, which gave trisulfonamides **11** after deprotection. Final ring closure was achieved by treatment of **11** again by using one equivalent of either dibromide **6** or dimesylate **8**. Ring closure with 1,4-dichloro-2-butyne gave extremely low yields and so was not normally undertaken (Scheme 3).

**Scheme 4:**
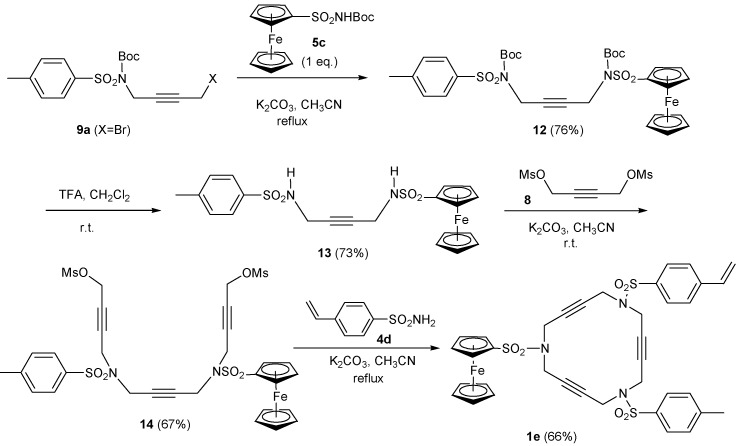
Synthesis of macrocycle **1e**.

The synthesis of macrocycle **1e** [39] containing three different aryl units required a modified pathway and was prepared as outlined in Scheme 4. Condensation of bromosulfonamide derivative **9a **with one equivalent of Boc-protected ferrocenylsulfonamide **5c** using potassium carbonate in refluxing acetonitrile led to a 76% yield of compound **12**. The elimination of Boc groups in compound **12** and subsequent treatment with an excess of 1,4-bis(methanesulfonyloxy)-2-butyne **8** resulted in the isolation of derivative **14 **with a 67% yield. The reaction of **14** with 4-vinylbenzenesulfonamide **4d** was straightforward and led to the formation of macrocycle **1e** featuring three different aryl substituents.

**Scheme 5:**
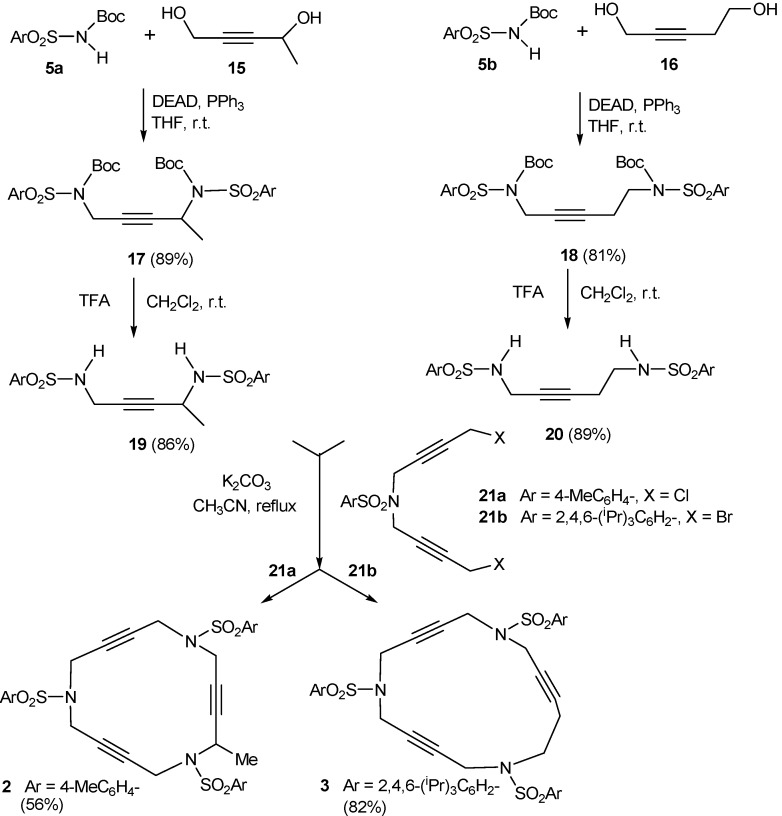
Synthesis of macrocycles **2** and **3**.

Furthermore, a 15-membered azamacrocycle containing a methyl group in one of the propargylic positions (**2**) [41] and a 16-membered macrocycle (**3**) [41] (see Figure 1) were also prepared following the synthetic pathway outlined in Scheme 5.

The main difference between these syntheses and those described above is the use of the Mitsunobu reaction. The whole synthesis started with the preparation of compounds **17 **and **18** by alkylation of Boc-protected sulfonamides **5a **and **5b** with 0.5 equivalents of the corresponding diol 2-pentyne-1,4-diol **15** or 2-pentyne-1,5-diol **16** under Mitsunobu reaction conditions. This made it possible to work with the diol derivatives to introduce the first alkyne chain under neutral alkylation conditions and avoided the elimination reactions that can take place in basic alkylation conditions in haloderivatives of compound **16**. The elimination of the Boc groups in compounds **17 **and **18** and the subsequent treatment with one equivalent of diacetylenic derivative **21a **and **21b** [47] respectively resulted in the isolation of macrocyles **2 **and **3 **with good overall yields.

**Figure 2:**
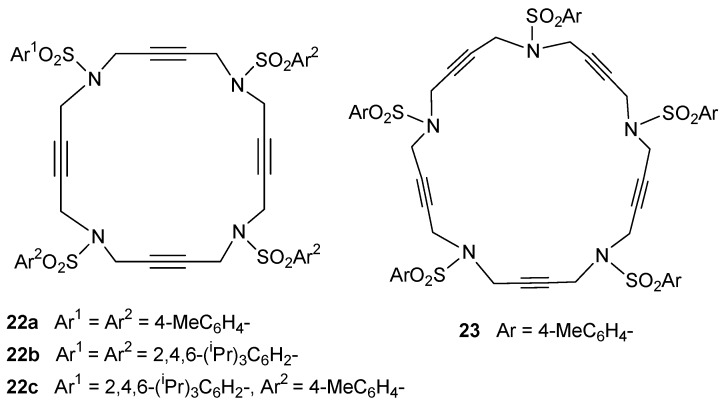
Tetra- and pentaacetylenic azamacrocycles.

The family of N-containing polyalkyne macrocycles was extended to higher order macrocycles, namely compounds of type **22** and **23** containing 4 or 5 triple bonds (and N) respectively (Figure 2).

**Scheme 6:**
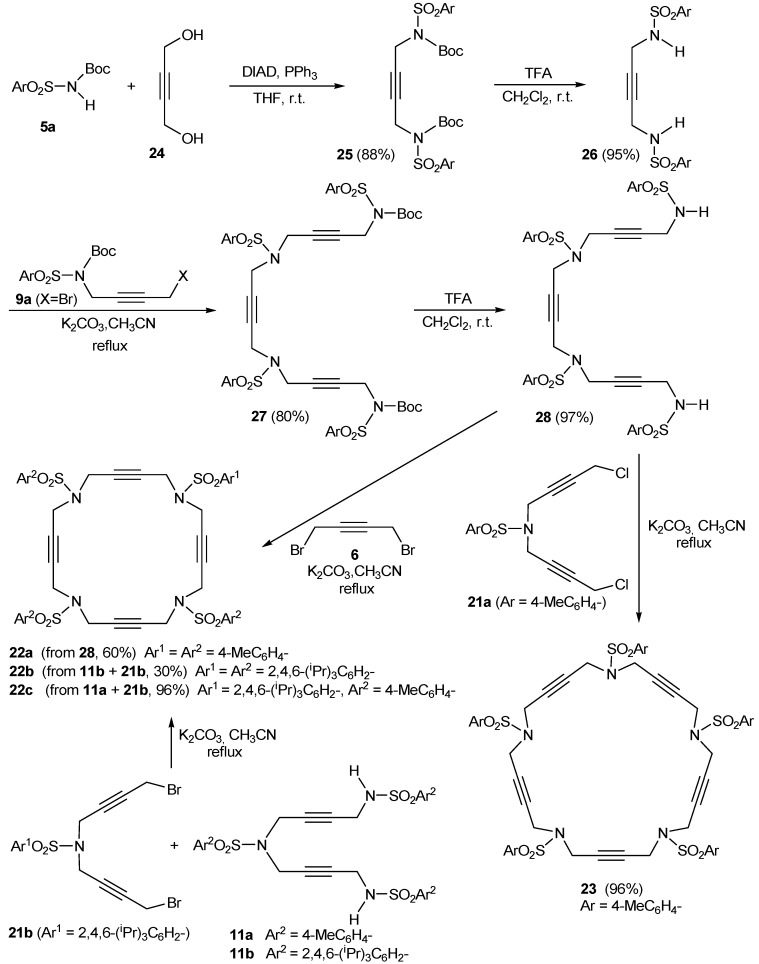
Synthesis of macrocycles **22a-c** and **23**.

The 20 and 25-membered macrocycles **22** [43] and **23** [43] were prepared following the synthetic pathway outlined in Scheme 6. One of the variants of macrocycles **22** is the nature of the aryl units on the periphery, analogously to the situation commented regarding the 15-membered macrocycles. Firstly, two macrocycles, **22a** and **23**, containing the same aryl unit, *p*-tolylsulfonamide, attached to all of the sulfonamides were prepared. The whole synthesis started with the preparation of compound **25** from a reaction of Boc-protected *p*-tolylsulfonamide **5a** and 0.5 equiv. of 2-butyne-1,4-diol **24** under Mitsunobu reaction conditions. The elimination of the Boc groups in compound **25** and the subsequent treatment with two equiv. of bromo derivative **9a** resulted in the isolation of compound **27 **with an 80% yield. The elimination of the Boc groups with the same reaction conditions as before (TFA in CH_2_Cl_2_) gave intermediate **28**. Compound **28**, which already contains three acetylenic chains and four identical sulfonamide units, was the key intermediate for the preparation of both macrocycles. Cyclization of **28 **with 1,4-dibromo-2-butyne in the presence of K_2_CO_3_ as a base afforded a 60% yield of macrocycle **22a**. When intermediate **28 **was condensed with the dichloro derivative **21a**, the 25-membered pentaacetylenic macrocycle **23 **was obtained in almost quantitative yield. Macrocycles **22b** and **22c** containing 2,4,6-triisopropylphenyl in all or some of the arylsulfonamide moieties, respectively, were prepared by an alternative synthetic route (Scheme 6). Condensation between dibromo derivative **21b** and trisulfonamide **11a **afforded a 96% yield of macrocycle **22c**. Reaction of **11b** with **21b** gave macrocycle **22b** containing four units of 2,4,6-triisopropylphenyl.

### 2.2. Preparation of enediyne azamacrocycles

**Figure 3:**
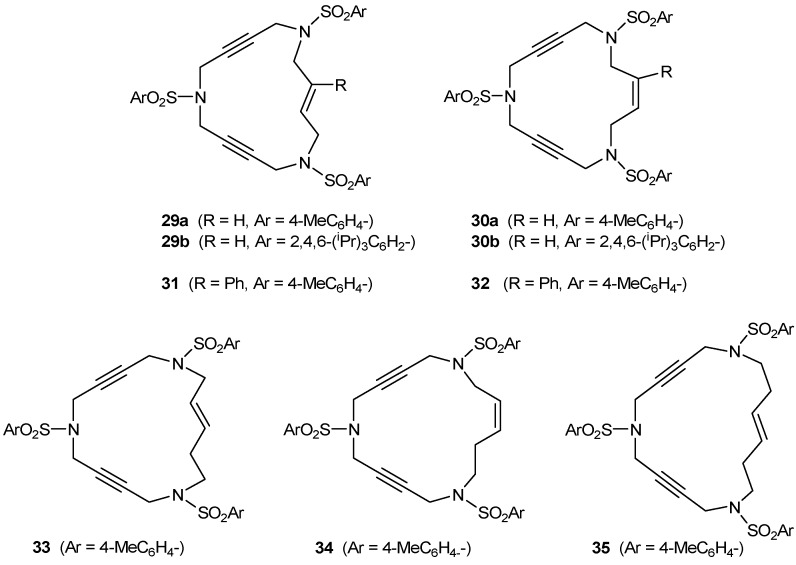
Enediyne azamacrocycles.

Since the [2+2+2] cycloaddition can also take place between two alkynes and one alkene as C_2_ sources, we were also interested in preparing enediyne macrocycles. The enediyne macrocycles which were prepared to test in the [2+2+2] cycloaddition reaction are represented in Figure 3. Structural variations were introduced in order to have double bonds of different stereochemistry (*trans***29**, **31**, **33**, **35** or *cis***30**, **32**, **34**), a substituent on the double bond (Ph, **31** and **32**), and macrocycles of different order (15-membered **29**-**32**, 16-membered **33**-**34** and 17-membered **35** macrocycles).

**Scheme 7:**
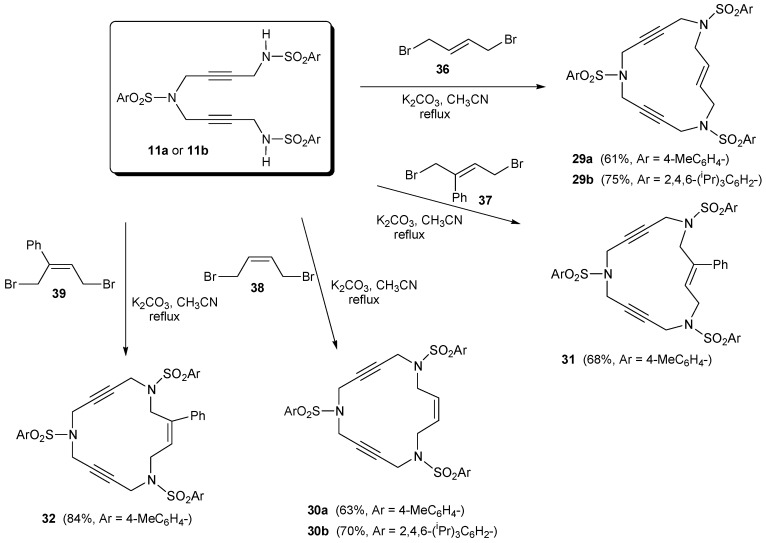
Synthesis of 15-membered enediyne macrocycles **29**-**32**.

The 15-membered enediyne macrocycles **29** [39], **30** [39], **31** [41] and **32** [41] were prepared starting from a common intermediate, the trisulfonamide derivative **11**, which reacted with the corresponding dibromo allylic derivative to afford the closed system (Scheme 7). This intermediate **11** contains either 4-methylphenyl (**11a**) or 2,4,6-triisopropylphenyl (**11b)** as the aryl units. Ring closure was achieved by treatment of **11** with one equivalent of either (*E*) or (*Z*)-1,4-dibromo-2-butene derivatives **36-39** in the presence of K_2_CO_3_ in refluxing acetonitrile.

**Scheme 8:**
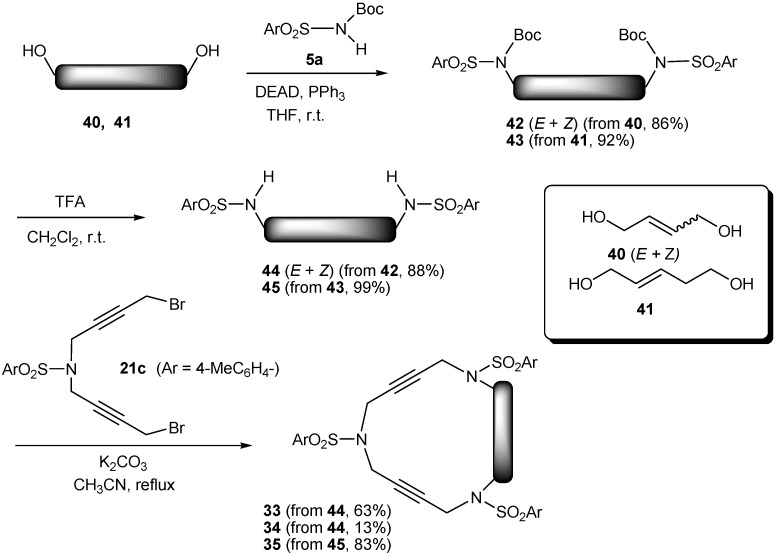
Synthesis of 16- and 17-membered enediyne macrocycles **33**, **34** and **35**.

In the case of enediynes **33-35** [41], in order to avoid β-elimination of the homoallylic position of the corresponding dibromo derivative in the ring closure when treated with base (K_2_CO_3_), the synthetic pathway applied was similar to the one in Scheme 5 for triacetylenic macrocycles **2 **and **3** (Scheme 8). We took Mitsunobu reaction conditions as the key to this pathway. The alkylation of N-Boc protected sulfonamide **5a** with diols **40 **and **41** under neutral Mitsunobu conditions afforded derivatives **42 **and **43** in excellent yields. After removal of the Boc groups with trifluoroacetic acid, ring closure was achieved by reaction with dibromo **21c **to give azamacrocycles **33-35**. Commercially available diol **40 **was a 4.5:1 mixture of *trans* and *cis* isomers and all the reactions of Scheme 8 were performed with this inseparable mixture. In the last step, macrocycles **33** (*trans*) and **34 **(*cis*) were separated by column chromatography. The *trans* was obtained with a 63% yield and the *cis* with a 13% yield.

## 3. [2+2+2] Cycloaddition Reactions

### 3.1. Cycloaddition reactions of tri-, tetra-, and pentaacetylenic azamacrocycles

Our investigation into cycloisomerization in the macrocyclic systems cited here began at a time of burgeoning interest in [2+2+2] cycloaddition reactions [1,2,3,4,5,6,7,8,9,10,11,12,13]. Our first aim was to test some of the metals that had proved to be more effective for related intra- and intermolecular cycloadditions in a macrocyclic scaffold (Table 1). Palladium complex Pd(PPh_3_)_4_ was the first metal of choice [48,49]. When **1a** was treated with catalytic amounts of Pd(PPh_3_)_4_, the reaction failed and it was necessary to use 1.1 equiv of Pd in refluxing toluene to obtain triazaindane **46a** in 54% yield (Entry 1, Table 1). If the reaction was run in THF and at room temperature, the macrocycle formed a highly stable Pd(0) complex in which the three triple bonds of **1a** were responsible for the complexation [38]. The same behaviour was observed for macrocycles **1b-1e**, all requiring stoichiometric quantities of palladium to afford cycloaddition products in moderate yields (Entries 2-5, Table 1). 

**Table 1:** Cycloisomerization reactions of macrocycles **1, 2**, and **3**. 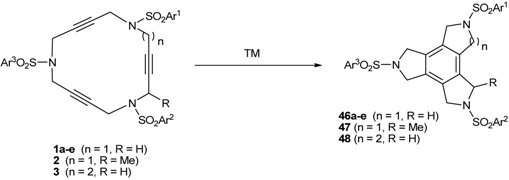

Entry	MCC	Catalyst(% molar)	Reaction conditions	Product	Yield (%)	Ref.
1	**1a**	Pd(PPh_3_)_4 _(110)	toluene, reflux, 22 h	**46a**	54	[38]
2	**1b**	Pd(PPh_3_)_4 _(110)	toluene, reflux, 24 h	**46b**	54	[39]
3	**1c**	Pd(PPh_3_)_4 _(110)	toluene, reflux, 24 h	**46c**	65	[39]
4	**1d**	Pd(PPh_3_)_4 _(110)	toluene, reflux, 24 h	**46d**	54	[39]
5	**1e**	Pd(PPh_3_)_4 _(110)	toluene, reflux, 24 h	**46e**	45	[39]
6	**1b**	CpCo(CO)_2_ (5)	decane, 140 ºC, 3.5 h	**46b**	44	[39]
7	**1b**	CpCo(CO)_2_ (100)	decane, 140 ºC, 1 h	**46b**	88	[39]
8	**1b**	Grubbs’ cat.^a^ (7)	toluene, reflux, 22 h	**46b**	36	[39]
9	**1d**	Grubbs’ cat.^a^ (7)	toluene, reflux, 22 h	**46d**	42	[39]
10	**1b**	Grubbs’ cat.^a^ (20)	toluene, reflux, 22 h	**46b**	36	[39]
11	**1a**	RhCl(CO)(PPh_3_)_2 _(5)	toluene, 65 ºC, 24 h	**46a**	88	[39]
12	**1b**	RhCl(CO)(PPh_3_)_2 _(5)	toluene, 65 ºC, 18 h	**46b**	96	[39]
13	**1d**	RhCl(CO)(PPh_3_)_2 _(5)	toluene, 65 ºC, 24 h	**46d**	89	[39]
14	**1b**	RhCl(CO)(PPh_3_)_2 _(1)	toluene, 65 ºC, 72 h	**46b**	80	[39]
15	**1a**	RhCl(PPh_3_)_3_ (10)	toluene, r.t., 1.5 h	**46a**	84	^b^
16	**2**	RhCl(PPh_3_)_3_ (10)	toluene, r.t., 26 h	**47**	91	^b^
17	**2**	RhCl(PPh_3_)_3_ (10)	toluene, 60 ºC, 24 h	**47**	99	[41]
18	**3**	RhCl(PPh_3_)_3_ (5)	toluene, 90 ºC, 28 h	**48**	81	[41]

^a^ Grubbs’ catalyst = ((PCy_3_)_2_Cl_2_Ru=CH-Ph). ^b^ Unpublished results.

Next, macrocycle **1b** was treated with a catalytic amount of cyclopentadienylcobalt dicarbonyl (CpCo(CO)_2_). The utility of the CpCo(CO)_2_ system as a catalyst for this chemistry was initially revealed by Vollhardt [1]. When the reaction was run with a 5% molar of CpCo(CO)_2_ in *n*-decane at 140 ºC for 3.5 hours a 44% yield of compound **46b** was obtained (Entry 6, Table 1). Using a stoichiometric amount of the cobalt complex, the yield of **46b** was improved to 88% (Entry 7, Table 1). Since stoichiometric quantities of Co^I^ complex were still required to obtain high yields, we turned our attention to other transition metal complexes. Ruthenium complexes such as Grubbs’ catalysthave been used as catalysts for the cyclotrimerization of alkynes [50,51,52]. Macrocycles **1b **and **1d** were treated with a catalytic amount of bis(tricyclohexylphosphane)benzylidene ruthenium(II) dichloride in refluxing toluene to afford the corresponding triazatrindanes **46b** and **46d** in moderate yields (36% and 42%, respectively) (Entries 8 and 9, Table 1). In the case of macrocycle **1b**, 20% of Grubbs’ catalyst was also tested, but the yield of **46b** did not improve (Entry 10, Table 1). On the other hand, rhodium complexes, such as Wilkinson’s catalyst [53,54,55], are also known to be effective for the synthesis of polysubstituted benzenes from alkynes [1,2,3,4,5,6,7,8,9,10,11,12,13]. Since chlorocarbonylbis(triphenylphosphane)rhodium(I) was available in our laboratory, this was tested in macrocycle **1a**. Using catalytic amounts of RhCl(CO)(PPh_3_)_2_ (5% molar) in toluene at 65 ºC, compound **46a** was obtained in 88% yield (Entry 11, Table 1). Similarly, the cycloisomerization of macrocycles **1b **and **1d** was run and in both cases the yields were high (Entries 12 and 13, Table 1). An attempt to reduce the catalytic amount of Rh(I) complex to 1% molar in the cycloaddition of **1b** did not prove to be as successful as when using 5% molar. After three days, the yield of **46b **was 80% and a 17% yield of starting material was recovered (Entry 14, Table 1). Wilkinson’s catalyst is also highly active for these macrocyclic systems and in the case of macrocycle **1a**, the reaction took place at room temperature using a 10% molar of the complex (Entry 15, Table 1). These results led us to conclude that rhodium is the most suitable of the transition metals tested as it permitted us to work with catalytic quantities and mild reaction conditions. We therefore used this metal preferentially in later experiments.

The next step in our investigation was to study the effect of slight structural variations on the macrocycle scaffold. The incorporation of a methyl group in the propargylic position (macrocycle **2**) did not seem to encumber the reaction. The reaction was run in toluene at room temperature for 26 hours giving a 91% yield (Entry 16, Table 1). By heating the reaction to 60 ºC for 24 hours this yield was improved to 99%. (Entry 17, Table 1). In the case of 16-membered azamacrocycle **3** it was necessary to heat the reaction to 90 ºC to obtain an 81% yield of **48**, showing that the formation of a 5,5,6-tetrafused structure is more difficult than the formation of 5,5,5-tetrafused system.

Another contribution that we have made in this area is to test other rhodium-based catalytic systems in order to evaluate their efficiency. All of these experiments were performed with macrocycle **1b**, whose arylic units of 2,4,6-triisopropylphenyl confer solubility both to the initial macrocycle and the cycloisomerization product (Table 2). Rhodium complexes stabilized by *N*-heterocyclic carbene (NHC) ligands have the advantages of being electronically versatile, easy to handle, and easily synthesized. In addition, unlike rhodium complexes stablized by phosphanes, they are not prone to oxidation and so the reactions can be run in aerobic conditions. Therefore, two rhodium complexes of *N*-heterocyclic carbenes were also tested for intramolecular [2+2+2] cycloaddition reactions of our closed systems: [RhCl(I*^i^*Pr)(cod)] (I*^i^*Pr = 1,3-diisopropylimidazolin-2-ylidene, cod = 1,5-cyclooctadiene) and [RhCl(IMes)(cod)] (IMes = 1,3-dimesitylimidazolin-2-ylidene). Macrocycle **1b **was treated with 5% molar of [RhCl(I*^i^*Pr)(cod)] in dichloromethane at room temperature to afford a 90% yield of **46b **after 7 days of reaction (Entry 1, Table 2). The reaction time was considerably decreased by switching to toluene and heating at 50 ºC (Entry 2, Table 2). Complex [RhCl(IMes)(cod)] was also able to promote the cycloaddition but needed more vigorous heating (Entry 3, Table 2). This fact may possibly be explained by the greater steric hindrance introduced by the mesityl substituents in the NHC [42].

Another aspect that we investigated was the recovery and reuse of the catalytic system. In collaboration with Muzart *et al.* we used molten tetra-*n*-butylammonium bromide to see whether this would be an efficient media for the [2+2+2] cycloaddition of macrocycle **1b **that might facilitate the recovery and reuse of the catalyst. Using a 5% molar of Wilkinson’s catalyst, an 80% yield of **46b **was obtained (Entry 4, Table 2). Furthermore, this molten salt was found to be a good immobilizing agent for the Wilkinson’s catalyst enabling recycling. The Rh complex and *n*-Bu_4_NBr mixture was dried under vacuum and reused although considerably reduced yields were obtained (39% and 24% yield in two successive recycles) (Entries 5 and 6, Table 2). With an increase in the initial amount of catalyst to 10% molar, the result of the first run was similar to that using a 5% molar (Entry 7, Table 2) although the recycling was more efficient (61%) (Entry 8, Table 2). In the same molten salt medium we tested PdCl_2_ as a catalyst. Even though in common organic solvents equimolar amounts of palladium catalyst were required to cyclize triacetylenic azamacrocycles **1** (Entries 1-5, Table 1), we decided to test the catalytic system PdCl_2_/*n*-Bu_4_NBr using only a 10% molar of PdCl_2_ at 130 ºC for 24 h. Surprisingly, compound **46b** was obtained in an 86% yield (Entry 9, Table 2) and recycling gave a moderate 36% yield (Entry 10, Table 2). When this catalytic system was used, palladium nanoparticles were identified by means of transmission electron microscopy (TEM) and energy dispersive X-ray spectroscopy (EDX), which are presumably the active catalytic species [40].

**Table 2:** Cycloisomerization reactions of macrocycle **1b **with different catalytic systems. 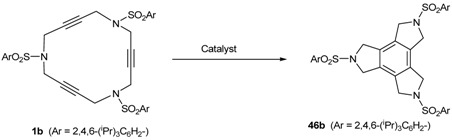

Entry	Catalyst (% molar)	Reaction conditions	Yield of 46b (%)	Ref.
1	RhCl(I*^i^*Pr)(cod)^a^ (5)	CH_2_Cl_2_, r.t., 7d	90	[42]
2	RhCl(I*^i^*Pr)(cod)^a^ (5)	toluene, 50ºC, 48h	98	[42]
3	RhCl(IMes)(cod)^b^ (5)	toluene, 90ºC , 24h	97	[42]
4	RhCl(PPh_3_)_3_ (5)	*n*-Bu_4_NBr,130ºC, 7h	80	[40]
5	Recycling entry 4	24h	39	[40]
6	Recycling entry 5	24h	24	[40]
7	RhCl(PPh_3_)_3_ (10)	*n*-Bu_4_NBr,130ºC, 3h	82	[40]
8	Recycing entry 7	24h	61	[40]
9	PdCl_2_ (10)	*n*-Bu_4_NBr,130ºC, 24h	86	[40]
10	Recycling entry 9	24h	36	[40]

^a^ [RhCl(I*^i^*Pr)(cod)], I*^i^*Pr = 1,3-diisopropylimidazolin-2-ylidene, cod = 1,5-cyclooctadiene; ^b^ [RhCl(IMes)(cod)], IMes = 1,3-dimesitylimidazolin-2-ylidene.

**Scheme 9:**
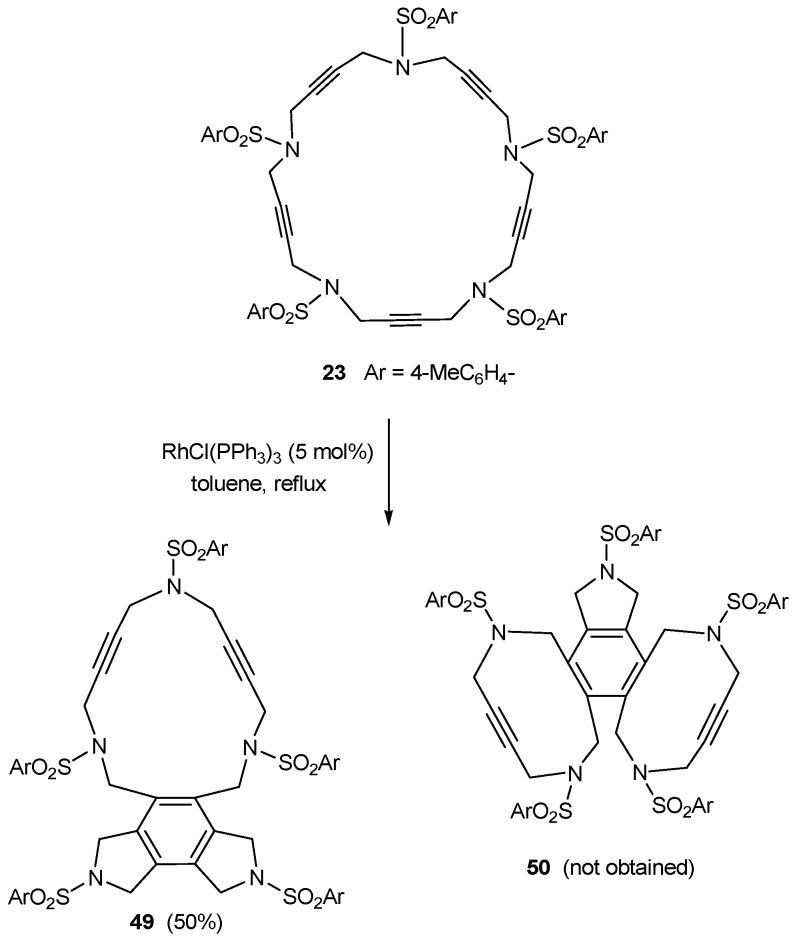
Cycloaddition of azamacrocycle **23**.

Wilkinson’s catalyst (RhCl(PPh_3_)_3_) was also selected for the cycloisomerization of 20- and 25-membered azamacrocyles **22** and **23**. When 20-membered macrocycles **22a-c **were treated with RhCl(PPh_3_)_3_ in refluxing toluene, no reaction took place. In all three cases, starting materials together with decomposition products were obtained. A stoichiometric amount of CpCo(CO)_2_ was also tested. The macrocycle was refluxed in toluene and the solution was heated by light irradiation. However, this reaction also failed and the starting macrocycle was recovered. In the case of the 25-membered ring **23**, there are two possible ways of cyclization, namely cycloaddition between three consecutive triple bonds to afford compound **49** and cycloaddition between non-consecutive triple bonds to afford compound **50**. Therefore, when 25-membered macrocycle **23** was treated with a catalytic amount of rhodium complex, the cyclotrimerized compound **49** resulting from the reaction of three contiguous alkynes was obtained as the only product of the process (Scheme 9). The lack of the reactivity of the 20-membered azamacrocycles **22** was explained by DFT calculations [43]. This theoretical study revealed that there are two main factors contributing to the lack of reactivity of the 20-membered macrocycle: firstly, macrocycle **22** has a more stable and delocalized HOMO orbital, and secondly, the formation of a strained ten-membered ring during the cyclotrimerization of **22**. These two factors increase the free energy barriers of the rate-determining step and make the intramolecular cyclotrimerization of the 20-membered azamacrocycles more difficult.

### 3.2. Cycloaddition reactions of enediyne azamacrocycles

Cycloaddition of two molecules of alkyne with an alkene is a straightforward route to substituted cyclohexadiene derivatives, which are important components for Diels-Alder reactions. Therefore, the cycloisomerization of enediyne azamacrocycles opens the door to obtain tetrafused cyclohexadienes with high functionality in a single step. The synthetic versatility of such a transformation becomes more interesting as it proceeds with total stereoselectivity with respect to the stereochemistry of the original double bond of the macrocyclic compound. Given this, we decided to study the cycloisomerization process in the series of enediyne azamacrocycles which we have prepared (**29-35** of Figure 3) (Table 3).

The first cases tested were 15-membered macrocycles **29** and **30** having a *trans* and a *cis* olefin respectively. Using a 5% molar of RhCl(CO)(PPh_3_)_3_ in toluene at 90 ºC gave high yields of the cycloisomerized compounds **51 **and **52** (Entries 1-4, Table 3). No side reactions of the cyclohexadiene system such as aromatization or further cycloadditions took place. The reaction proceeded with total stereoselectivity and initial stereochemistry of the macrocyclic double bond was maintained during the cycloaddition process. This experimental finding is consistent with the common mechanism proposed for this kind of cycloadditions in which two alkyne groups undergo initial coupling and subsequent incorporation of the olefin may then occur either by an insertion process or a Diels-Alder reaction.

**Table 3:** Cycloaddition of enediyne azamacrocycles **29**-**35**. 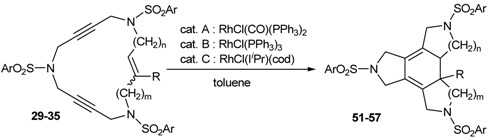

Entry	MCC	Reaction conditions	Product	Yield (%)	Ref.
1	**29a**	cat. A (5% molar), 90 ºC, 24 h	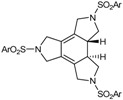	98	[39,41]
			**51a** (Ar = 4-MeC_6_H_4_-)		
2	**29b**	cat. A (5% molar), 90 ºC, 24 h	**51b** (Ar = 2,4,6-(^i^Pr)_3_C_6_H_2_-)	80	[39]
3	**30a**	cat. A (5% molar), 90 ºC, 24 h	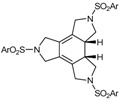	79	[39]
			**52a** (Ar = 4-MeC_6_H_4_-)		
4	**30b**	cat. A (5% molar), 90 ºC, 24 h	**52b** (Ar = 2,4,6-(^i^Pr)_3_C_6_H_2_-)	68	[39]
5	**29b**	cat. B (5% molar), 90 ºC, 24 h	**51b** (Ar = 2,4,6-(^i^Pr)_3_C_6_H_2_-)	80	[39]
6	**29b**	cat. C (5% molar), 50 ºC, 3 d	**51b** (Ar = 2,4,6-(^i^Pr)_3_C_6_H_2_-)	98	[42]
7	**31**	cat. B (5% molar), reflux, 24 h	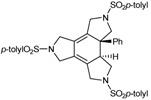	95	[41]
			53		
8	**32**	cat. B (5% molar), reflux, 24 h	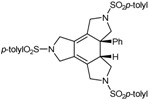	71	[41]
			54		
9	**33**	cat. B (10% molar), 80 ºC, 5 h	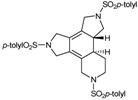	90	[41]
			55		
10	**34**	cat. B (10% molar), 80 ºC, 5 h	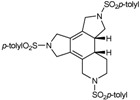	87	[41]
			56		
11	**35**	cat. B (10% molar), 60 ºC, 4 h	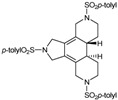	98	[41]
			57		

In order to see whether the Wilkinson’s catalyst also promotes the cycloisomerization process, macrocycle **29b** was treated with 5% molar of RhCl(PPh_3_)_3_ in toluene at 90 ºC. Compound **51b** was obtained in an 80% yield, demonstrating that Wilkinson’s catalyst exhibits a similar efficiency with respect to RhCl(CO)(PPh_3_)_2 _(Entry 5, Table 3). Macrocycle **29b **was also cycloisomerized using the rhodium-N-heterocyclic carbene complex [RhCl(I*^i^*Pr)(cod)] which after 3 days at 50 ºC in toluene gave a 98% yield of **51b** (Entry 6, Table 3) [42].

The effect of variations on the macrocycle scaffold, either substituents on the double bond or larger cavities, was then tested on the [2+2+2] cycloaddition of enediyne macrocycles. Harsher reaction conditions were required to cycloisomerize macrocyclic enediynes containing a phenyl substituent on the double bond (**31 **and **32**). Refluxing toluene was necessary whereas 90 ºC was sufficient for the non-substituted macrocycles **29 **and **30 **(Entries 7 and 8, Table 3). In order to study the scope of the methodology, we chose different macrocycles (**33**, **34 **and **35**) whose later cycloisomerization led to various fused tetracycles such as 5,5,6- and 5,6,6-ring systems. As a general trend we observed that for enediyne macrocycles, the formation of 5,6,6-membered rings fused to the cyclohexadienic core (product **57**) was much faster than the formation of 5,5,6-ring system (products **55** and **56**), which in turn was faster than the formation of the 5,5,5-tetrafused structures (products **51 **and **52**) (Entries 9-11, Table 3) [41]. Although there was a certain tendency to the formation of larger rings, which gave faster reactions, all the macrocycles afforded fused tetracycles unlike in other methods of synthesis, where the failure to construct 5,5,5- has been attributed to ring constraint [56].

The enantioselective cycloaddition of enediynes **29**, **31**-**35** (Figure 3) using a chiral rhodium complex which would lead to chiral cycloadducts was the next aspect to be evaluated. There are only two reported studies of enantioselective cycloaddition of open-chain enediynes using chiral catalysts to afford enantioenriched cyclohexadienes. The two studies used chiral rhodium complexes and the choice of chiral ligands, as well as the nature of the tether between unsaturations, was very important to obtain good yields and high enantiomeric excesses [57,58].

The study of the enantioselective version of the cycloaddition reaction was undertaken with macrocycles **29a **and **33** (Table 4). The first chiral rhodium complex tested was the commercially available (bicyclo[2.2.1]hepta-2,5-diene)[2*S*,3*S*]-bis(diphenylphosphino)butane rhodium(I) perchlorate. The best reaction conditions found to obtain the high ratio yield/enantiomeric excess was toluene as a solvent at 65 ºC for one day for the two macrocycles **29a **(Entry 1, Table 4) and **33 **(Entry 2, Table 4). In the two cases, only moderate enantiomeric excesses were obtained. In order to improve these results, a chiral bidentate ligand, *N*-phosphino *tert*-butylsulfinamide (PNSO) prepared by Prof. Riera’s group was tested in a collaborative study with our own group. 

Macrocycle **29a **was treated with *N*-phosphino *tert*-butylsulfinamide rhodium complex in CH_2_Cl_2_ at room temperature. After 28 h of reaction the yield of **51a **was 77% and the ee was 48% (Entry 3, Table 4). When dichloromethane was substituted for toluene the reaction time was reduced considerably from 28 h to 5.5 h and a 79% yield of **51a **with a slightly improved 50% of ee was obtained (Entry 4, Table 4). The later reaction conditions were applied for macrocycle **33**. In this case an excellent yield of 94% of **55** was obtained although the ee dropped to 7% (Entry 5, Table 4). The enantiomer formed with the PNSO/Rh complex was the opposite of that obtained in entries 1 and 2. Other experiments using [RhCl(COD)]_2_ and several chiral phosphines did not lead to better chirality induction than that described in Table 4.

**Table 4:** Enantioselective [2+2+2] cycloaddition reactions of **29a **and **33**. 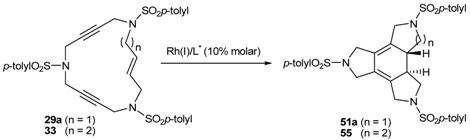

Entry	MCC	Catalyst (10% molar)	Reaction conditions	Product	Yield (%)	e.e (%)	Ref.
1	**29a**	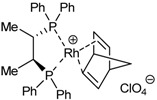	toluene, 65ºC, 24h	**51a**	95	44	[41,44]
2	**33**	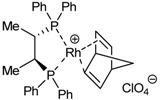	toluene, 65ºC, 24h	**55**	46	41	[41,44]
3^a^	**29a**	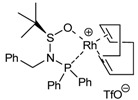	CH_2_Cl_2_, r.t., 28h	**51a**	77	48	[44]
4^a^	**29a**	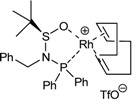	toluene, r.t., 5.5h	**51a**	79	50	[44]
5^a^	**33**	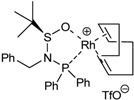	toluene, r.t., 5.5h	**55**	94	7	[44]

^a^ The enantiomer obtained in these cases was the opposite of that obtained in entries 1 and 2.

### 3.3. Cycloaddition reactions of triacetylene and enediyne azamacrocycles in the absence of transition metals

**Scheme 10:**
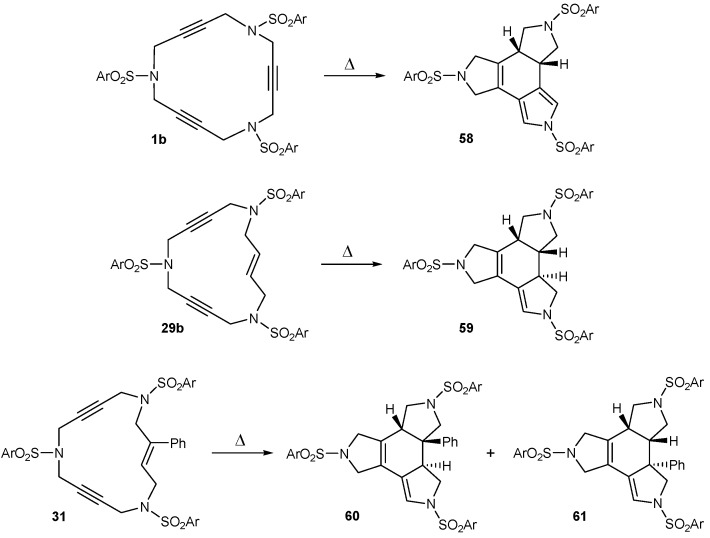
Thermally-induced cycloisomerizations.

The search for new catalysts on the [2+2+2] cycloaddition inside macrocyclic scaffolds has been one of the aims of the present project. During this investigation we found that our macrocyclic systems suffered a thermally induced transformation when the catalyst used was ineffective for the metal-catalyzed [2+2+2] cycloaddition. Three different 15-membered azamacrocycles, namely the triacetylenic **1b**, the (*E*)-enediynic **29b** and the (*E*)-enediynic bearing a phenyl substituent in the double bond **31**, were submitted to the thermal conditions giving rise to new cycloadducts in a stereoselective manner as shown in Scheme 10. 

The reaction conditions were varied in order to optimize the process and to find a mechanistic outcome for the overall transformation (Table 5). When the reaction was run without additives, only a moderate yield of the cycloadducts **58** and **59** was obtained after prolonged heating (Entries 1 and 2, Table 5). The yield increased substantially when adding an excess of 1,4-cyclohexadiene (1,4-CHD) (Entries 3-5, Table 5) and also moderately when switching the solvent from toluene to chlorobenzene (Entries 6 and 7, Table 5). 

**Table 5:** Thermal cycloisomerizations of macrocycles **1b**, **29b**, and **31**.

Entry	Substrate	Additive	Reaction conditions	Product	Yield (%)
1	**1b**	---	toluene, 110 ºC, 30 h	**58**	32
2	**29b**	---	toluene, 110 ºC, 6 d	**59**	45
3	**1b**	1,4-CHD	toluene, 110 ºC, 60 h	**58**	77
4	**29b**	1,4-CHD	toluene, 110 ºC, 6 d	**59**	78
5	**31**	1,4-CHD	toluene, 110 ºC, 11 d	**60+61**	81^a^
6	**1b**	---	chlorobenzene, 110 ºC, 30 h	**58**	62
7	**29b**	---	chlorobenzene, 110 ºC, 60 h	**59**	60

^a^ Combined yield of the two isomers, formed in equimolar quantities.

The role of radical species in the thermal transformation was clarified by means of an EPR study. This showed that benzyl radicals were formed in the cycloaddition in toluene without additives and that these radicals were found to be detrimental to the reaction. Experimental confirmation was obtained by adding benzoyl peroxide, which completely decomposed the starting material. Therefore the addition of radicals neutralizing agents such as 1,4-CHD or the use of a solvent which is less prone to homolytic cleavage such as chlorobenzene led to yield enhancement.

**Scheme 11:**
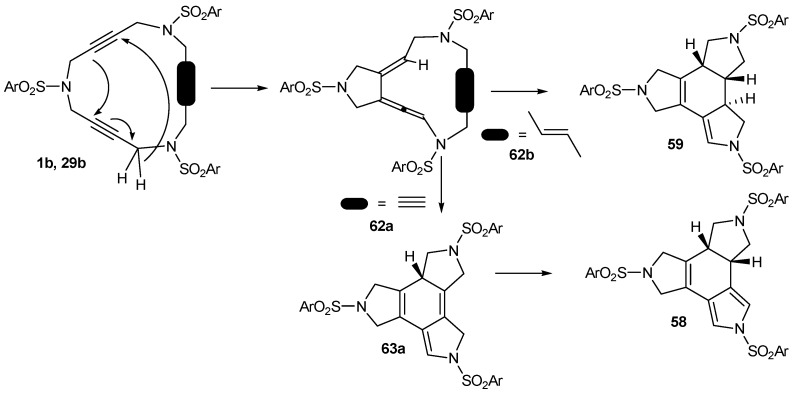
Mechanistic proposal for the thermally induced process.

A mechanistic proposal was made for the process based on a tandem intramolecular ene/Diels-Alder reaction (Scheme 11). The first step, an ene reaction which affords a vinylallene intermediate **62**, is followed up with a Diels-Alder reaction with the third unsaturation. If this is a triple bond (**62a**), the Diels-Alder reaction gives a 1,4-cyclohexadiene (**63a**) which suffers a proton rearrangement to yield **58**. If the third unsaturation is a double bond (**62b**), the Diels-Alder reaction directly yields **59**. In the case of **31**, the same mechanism accounts for the two products **60** and **61**, each arising from one of the two unequivalent alkynes participating as the ene or enophyle in the reaction. The proposed mechanism was backed up by DFT calculations which gave barriers that were confirmed experimentally by differential scanning calorimetry (DSC) analysis [59]. This mechanistic proposal has served as a model for Danheiser *et al.* [60,61] in their thermally induced intramolecular [2+2+2] cycloaddition reactions of triynes and cyanodiynes.

## 4. Concluding Remarks

The [2+2+2] cycloaddition reaction is a powerful tool to construct complex molecules in a one-step, atom economical procedure. The present work clearly shows the potential for this transformation inside macrocyclic scaffolds which give rise to fused tetracycles in a highly efficient manner. Various synthetic routes leading to aza-containing polyalkyne and enediyne macrocycles have been presented. The effectiveness of the cycloaddition reaction has been shown to be dependant not only on the nature of the catalyst system, but also on the size and morphology of the macrocycle. Furthermore, the mechanistic proposal for a thermally induced cycloisomerization which is not catalyzed by metals but that can also take place in the macrocycles has been described.
